# The Effectiveness of Disaster Risk Communication: A Systematic Review of Intervention Studies

**DOI:** 10.1371/currents.dis.349062e0db1048bb9fc3a3fa67d8a4f8

**Published:** 2014-08-22

**Authors:** Declan T Bradley, Marie McFarland, Mike Clarke

**Affiliations:** Public Health Agency and Queen's University Belfast, Belfast, Northern Ireland, United Kingdom; Belfast Health and Social Care Trust, Department of Pathology, Belfast, Northern Ireland, United Kingdom; Queen's University Belfast, Belfast, Northern Ireland, United Kingdom

## Abstract

Introduction: A disaster is a serious disruption to the functioning of a community that exceeds its capacity to cope within its own resources. Risk communication in disasters aims to prevent and mitigate harm from disasters, prepare the population before a disaster, disseminate information during disasters and aid subsequent recovery. The aim of this systematic review is to identify, appraise and synthesise the findings of studies of the effects of risk communication interventions during four stages of the disaster cycle. Methods: We searched the Cochrane Central Register of Controlled Trials, Embase, MEDLINE, PsycInfo, Sociological Abstracts, Web of Science and grey literature sources for randomised trials, cluster randomised trials, controlled and uncontrolled before and after studies, interrupted time series studies and qualitative studies of any method of disaster risk communication to at-risk populations. Outcome criteria were disaster-related knowledge and behaviour, and health outcomes. Results: Searches yielded 5,224 unique articles, of which 100 were judged to be potentially relevant. Twenty-five studies met the inclusion criteria, and two additional studies were identified from other searching. The studies evaluated interventions in all four stages of the disaster cycle, included a variety of man-made, natural and infectious disease disasters, and were conducted in many disparate settings. Only one randomised trial and one cluster randomised trial were identified, with less robust designs used in the other studies. Several studies reported improvements in disaster-related knowledge and behaviour. Discussion: We identified and appraised intervention studies of disaster risk communication and present an overview of the contemporary literature. Most studies used non-randomised designs that make interpretation challenging. We do not make specific recommendations for practice but highlight the need for high-quality randomised trials and appropriately-analysed cluster randomised trials in the field of disaster risk communication where these can be conducted within an appropriate research ethics framework.

## Introduction

A disaster is a “serious disruption of the functioning of a community or a society involving widespread human, material, economic or environmental losses and impacts, which exceeds the ability of the affected community or society to cope using its own resources.”^1^ Four stages of a ‘disaster cycle’ have been identified: Mitigation and prevention, preparedness, response, and recovery.^2^^,^ 3 Communication between authorities and the public about disasters occurs in all stages of the cycle, with different aims at each stage. Communication is a potentially valuable way of avoiding and reducing harm caused by disasters.

Risk communication aims to provide the public with information about the effects of an event, and how actions may affect the outcome of the event.^4^ Crisis and Emergency Risk Communication (CERC) is the use of risk communication in emergencies to inform the public about an event or issue to empower members of a community to protect themselves.^4^ In this review, we focus on CERC in the context of disasters, at all stages of the disaster cycle. Risk communication in disasters has historically been a one-way transfer of information from authorities to the public, rather than an interactive flow of information.^5^ Disaster risk communication may take place through many different channels, including some that have been recently developed or expanded. Potential channels of communication include face-to face conversations, telephone calls, group meetings, mass media such as television, tailored mass media such as reverse 911 services and interactive social media such as Twitter.

The effectiveness of risk communication interventions could be evaluated by assessing many possible outcomes. We chose to focus on knowledge, behaviour and incidence of health outcomes (e.g. injuries, deaths), which are particularly likely to be measured and reported, and also might be considered the most important outcomes.

As well as seeking to estimate the effects of different types of disaster risk communication, we aim to identify gaps in knowledge or evidence that would benefit from future research. Lastly, we aim to identify lessons from the literature that will help inform the design of future research.

## Methods

**Protocol and registration**

****This project was undertaken as part of one author's Masters in Public Health degree (DTB) and was not externally registered in advance. The review is reported according to the PRISMA statement (PRISMA checklist is shown in Appendix 1).

**Eligibility criteria and outcomes**

****The following intervention study types were included: Randomised trials, cluster randomised trials, quasi-randomised trials, controlled before-and-after studies, uncontrolled before-and-after studies, post-intervention only studies if the pre-intervention state could reasonably be assumed, interrupted time series and qualitative research.

The populations included were people or communities, before, during or following a disaster, who were at risk of being affected by a public health problem. Groups of people considered more vulnerable to disasters because of their language or other characteristics (e.g. disability) were included in the review. Communications must have been made from public authorities to communities or populations. Studies of communication with or between groups of professionals involved in disaster response were excluded from the review.

The interventions included were face-to-face, television, radio, Internet or telephone communication, or any other method of risk communication aimed at informing the public about a potential disaster situation to enable people to make informed choices that benefit their health on or after 1 January 2000. Risk communication interventions were aimed at producing desired knowledge, behavioural or health outcomes. This time limit was chosen because of recent development in electronic communications.

The United Nations International Strategy for Disaster Reduction definition of disaster was adhered to.^1^ The HIV pandemic was excluded from this review because an extensive body of literature exists for this specific topic, which has been reviewed several times in recent years. If it was not clear from the article whether a study took place in the context of a disaster or if the intervention was not described in sufficient detail to be assessed, the study was categorised as 'unclear'.

The outcomes included in this review were: 1. Incidence of health-related events related to the disaster/possible disaster; 2. Health-related behaviour (self-reported or observed) relating to the disaster/possible disaster; 3. Health-related knowledge about the disaster/possible disaster.

**Information Sources**

We searched the following databases: Cochrane Central Register of Controlled Trials (CENTRAL; 2000 to present); Embase (Ovid, 2000 to present); MEDLINE (Ovid, 2000 to present); PsycInfo (Ovid, 2000 to present); Sociological Abstracts (Proquest, 2000 to present) and Web of Science (Web of Knowledge, 2000 to present). These online databases were searched for relevant articles on 18 June 2013. Searches were not limited by language or country of origin but were limited to manuscripts published on or after 1 of January 2000. The search strategy was constructed from a combination of disaster-related search terms and communication-related search terms. Outcomes were not specified in the search in order to avoid excluding relevant studies. Database selection and the principles of the search strategy were discussed with a subject librarian.

Full search strategy for all databases is shown in Appendix 2.

Further articles were identified from references of potentially relevant studies. The websites of the Centers for Disease Control and Prevention, Public Health England, European Centre for Disease Control, the World Health Organisation, The United Nations Office for Disaster Risk Reduction and the World Bank were searched for documents relevant to disaster communication. The New York Academy of Medicine's Grey Literature Report was also searched for "disaster communication."

**Data collection and assessment**

Search results were merged and duplicates removed. Two reviewers (DTB and MM) independently read the retrieved titles and abstracts and assessed them for relevance. Reports identified as potentially relevant by either reviewer were retrieved in full and independently assessed for inclusion by two reviewers (DTB and MM), with a discussion to resolve discordances. Published data were extracted by one reviewer (DTB) using a standardised form. No unpublished data were sought.

Data Items collected were: Disaster cycle phase (mitigation, preparedness, response or recovery); Disaster type (infectious disease, natural or man-made); Study type; Geographical setting; Population characteristics and numbers; Randomisation method if applicable; Allocation method if applicable; Blinding; Attrition if applicable; Details of intervention; Outcomes reported; Results.

Study-level assessment for bias was performed with the Cochrane Collaboration’s ‘Assessment for bias’ tool. The tool was not ideally suited to the non-randomised studies. The assessment tool was used to catalogue the characteristics of the study without attempting to make an overall summary categorisation of study bias as their very diverse natures, contexts and methods made most studies not directly comparable, even in terms of bias.

All relevant knowledge, behaviour and incidence outcomes were included in the review in the manner that they were originally reported. Due to the disparate nature of the studies, no formal assessment of risk of bias across studies was considered. No additional analyses were performed.

## Results

**Study selection**

The electronic searches yielded 5,224 unique articles. Of these, 100 were judged to be potentially relevant by at least one reviewer and were obtained in full. Of these 100 articles, 25 were judged to meet the inclusion criteria, and for a further six studies were judged not to clearly meet the inclusion criteria due to insufficient information. Two additional studies were found from references of other papers, and one additional paper providing extra information about an already included study was also identified (Figure 1). Full study characteristics and assessment for bias for included reports are shown in Appendix 3, references to excluded studies with reasons for exclusion are shown in Appendix 4 and references to studies for which an assessment could not be made are shown in Appendix 5.

**Review Flowchart:**
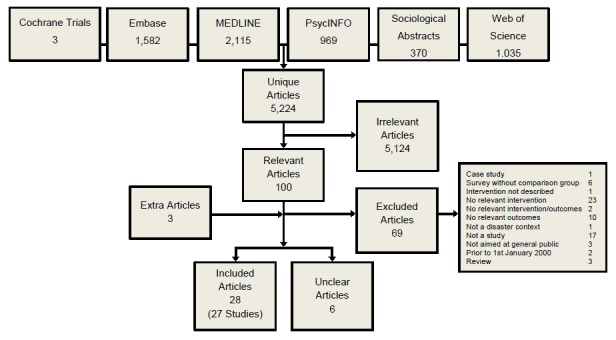
Flowchart showing results of systematic review process.

**Study Characteristics**

**Summary characteristics of included studies:**
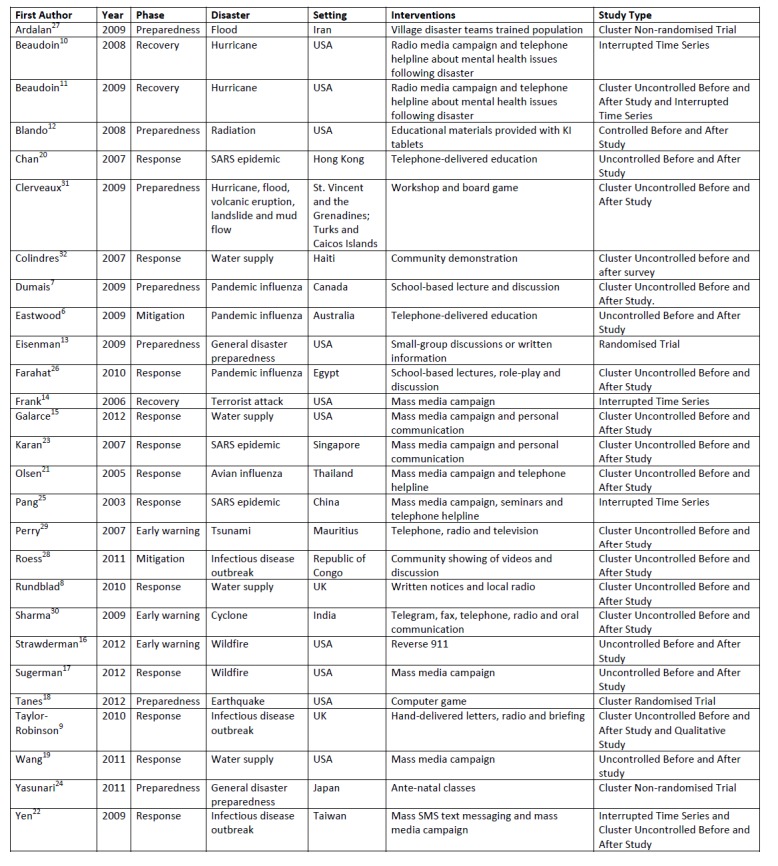

Summary characteristics of the studies are shown (Figure 2). One study took place in Australia,^6^ one in Canada,^7^ two in the United Kingdom^8^^,^ 9 and ten in the United States of America.^10^^,^ 11 ^,^ 12 ^,^ 13 ^,^ 14 ^,^ 15 ^,^ 16 ^,^ 17 ^,^ 18 ^,^ 19 One was conducted in Hong Kong,^20^ one in Thailand,^21^ one in Taiwan,^22^ one in Singapore,^23 ^one in Japan,^24^ and one in China.^25^ One study took place in Egypt,^26^ one in Iran,^27^ one in Republic of the Congo,^28^ one in Mauritius,^29^ one in India,^30^ one in Haiti^31^ and one took place on two Caribbean islands: St. Vincent and the Grenadines, and Turks and Caicos Islands.^32^

Two studies focused on general disaster preparedness,^13^^,^ 24 one focused on five natural hazards,^31^ two on hurricanes only,^10^^,^ 11 one on a cyclone,^30^ one on floods only,^27^ two on wildfires,^16^^,^ 17 one on earthquakes^18^ and one on a terrorist attack.^14^ Four studies dealt with water supply disasters.^8^^,^ 14 ^,^ 19 ^,^ 32 One dealt with radiation.^12^ The remaining studies focused on infectious disease disasters: Three studied pandemic influenza,^6^^,^ 7 ^,^ 26 three focused on the SARS epidemic,^20^^,^ 23 ^,^ 25 one on an outbreak of avian influenza,^21^ one on an epidemic of acute haemorrhagic conjunctivitis,^22^ one followed an outbreak of monkeypox^28^ and one dealt with an outbreak of meningococcal disease.^9^

There were several different types of communication method used in these studies. Mitigation and preparedness interventions used face-to-face group participation and education in eight studies,^6^^,^ 7 ^,^ 12 ^,^ 13 ^,^ 24 ^,^ 27 ^,^ 28 ^,^ 31 one of which included the use of a specially designed board game.^31^ Two early warning alerts used several communication channels^29^^,^ 30 and another used Reverse-911.^16^ Mitigation and preparedness studies that did not use face-to-face communication used a telephone intervention,^6^ and a computer game.^18^ The response phase interventions were multi-channel information campaigns (with different methods and balance of methods between studies)^8^^,^ 9 ^,^ 15 ^,^ 17 ^,^ 19 ^,^ 20 ^,^ 21 ^,^ 22 ^,^ 23 ^,^ 25 ^,^ 32 and a school-based participatory intensive education programme.^26^ The three recovery phase interventions used media campaigns to encourage members of the public to access telephone help lines for mental health problems and to undertake healthy behaviours following disasters.^10^^,^ 11 ^,^ 14

The objectives of the interventions were to improve the health knowledge and behaviour in relation to disasters, and to decrease the incidence of negative health events. The studies were chosen on that basis, and several studies reported additional outcomes that were outside the scope of this review. The knowledge, behaviour and incidence outcomes are described in detail in Appendix 3 (characteristics and results of included studies).

## Results of Individual Studies

**1 Effect of Risk Communication Interventions to Promote Disaster Mitigation and Preparedness**

**1.1 Communication to Promote Mitigation and Preparedness for Infectious Disease Disasters**

**1.1.1 Setting**

Two studies for pandemic influenza took place in high-income countries (Canada^7^ and Australia^6^), and one study for monkeypox took place in a low-income country (Republic of the Congo).^28^ Both studies of interventions for pandemic influenza took place in 2008, before the 2009 H1N1 pandemic. The study of an intervention for monkeypox took place in 2009, six years after an outbreak in Republic of the Congo, and subsequent to a serological survey that suggested that past infection was widespread. Monkeypox is a potentially fatal disease that is related to smallpox, incidence of which is thought to have increased following the discontinuation of the smallpox vaccine in 1980.^28^

**1.1.2 Participants**

The Australian study by Eastwood *et al.* of a pandemic influenza mitigation intervention included 1,166 individuals (62% female) who were contacted by telephone (58% response rate).^6^ Dumais et al. reported an educational intervention in a class of 35 female year 10 students in a private school in Canada.^7^ The intervention in Republic of the Congo was delivered on a much greater scale: approximately 23,860 people in 16 towns and villages received the intervention over a period of 90 days.^28^ Evaluation was by a survey of 271 people (57% male; mean age 33 years) before and after the intervention.^28^

**1.1.3 Description of Interventions**

The Eastwood et al. intervention for pandemic influenza was a simple telephone conversation, with a researcher delivering a short script explaining the nature and effects of an influenza pandemic.^6^ The Dumais *et al.* intervention was developed by conducting a pre-intervention survey of students' understanding of viruses and forming an education programme based on revealed insufficiencies.^7^ The intervention took place in an 80 minute session involving a lecture and discussion, and addressed shortfalls in knowledge about virus biology. The aim of this intervention was to improve understanding of viruses to facilitate response to risk communication about pandemic and epidemic influenza. The Roess *et al.* intervention was an outreach education programme in villages in the Republic of Congo.^28^ Small group sessions were conducted using two specially made educational videos and discussions about monkeypox in each village.

**1.1.4 Results**

All three studies were uncontrolled before and after studies. The Eastwood et al. study of pandemic influenza revealed that the Australian public reported themselves to be largely willing to comply with the public health measures proposed, such as self-quarantine and social distancing. Following the information intervention, they were even more likely to report their willingness to self-isolate (94.1% before to 97.5% after), avoid public events (94.2% to 98.3%) and postpone gatherings (90.7% to 97.2%).^6^ Participants in the Canadian study of influenza increased their knowledge scores between a pre- and post-intervention test (with a significant change in mean score from 0.2 to 1.3, where 2.0 was the maximum possible score).^7^ Roess *et al.* analysed their before and after study using paired analyses.^28^ Significant improvements were noted in outcomes related to recognising monkeypox and knowledge of how it is transmitted. The numbers of people who knew at least one symptom increased from 49% to 95%, named rash and fever as symptoms increased from 11% to 32%, and knew that the rash occurs on palms and soles increased from 14% to 51%. The numbers of people who knew that the disease could be transmitted by contact with an ill person increased 28% to 58%, by fomites (2% to 14%), by contact with an ill animal (24% to 64%) and contact with a dead animal (7% to 19%). There were significant improvements in four appropriate behavioural actions. Whilst there was a reduction in the number of who would eat or sell found rodent carcasses (eating, 33% to 16%; selling 7% to 3%), there was no reduction in the number who would eat or sell primate carcasses (eat, 11%; sell, 4%).^28^ The authors noted that the programme had shown effectiveness in increasing knowledge, and might encourage participants to seek healthcare if monkeypox was suspected, but suggested that future strategies should focus on prevention of risk behaviours. The design used in all three infectious disease mitigation or preparedness studies means that inferring causality from the reports is difficult. Alternative explanations for the improvements in knowledge and behaviour include an observer effect, which might influence self-reported behaviour in the study of monkeypox and the Australian study of pandemic influenza.^6^

**1.2 Communication to Promote Preparedness for Natural Disasters**

**1.2.1 Setting**

Five studies of preparedness for natural disasters were identified. Two studies took place in the USA,^13^^,^ 18 one in Japan,^24^ one in Iran^27^ and one on the Carribean islands of St. Vincent and the Grenadines, and Turks and Caicos.^31^ Two studies were aimed at general preparedness,^13^^,^ 24 one preparedness for flash floods,^27^ one for earthquakes,^18^ and one for five natural hazards.^31^

**1.2.2 Participants**

Eisenman *et al. *reported a randomised trial of 231 Latino people who lived in Los Angeles County, California, USA, of whom 187 completed the study (a per protocol analysis was presented; mean age was 37 years; both groups were approximately two thirds female).^13^ Yasunari et al. reported a cluster non-randomised trial at different hospital sites with 99 primiparous women (mean age 31 years; mean gestation 22.9 weeks) in the intervention group (of 993 who attended these sessions) and 104 primiparous women (mean age 31 years; mean gestational 24.7 weeks) in the control group (of 1,010 who attended the control sessions).^24^ Tanes reported a cluster randomised trial of 250 college students (62% male) from "a large Midwestern university".^18^^,^ 33 Students selected a time slot to attend for the intervention, and the time slots were randomly assigned to an intervention. Clerveaux *et al.* conducted an uncontrolled cluster before and after study with 42 grade 5 pupils, with mean age 11 years, from St. Vincent and the Grenadines, and 33 Grade 5 pupils, with mean age 10 years, from Turks and Caicos.^31^ Ardalan conducted a cluster non-randomised trial in two rural flood-prone areas of Golestan Provence, Iran using independent pre- and post-intervention samples in each area.^27^ The intervention group included 1,163 participants pre-intervention and 1,159 post-intervention. The control group included 1,200 pre-intervention and 1,210 post-intervention. The mean age for each group was between 27 and 29 years, and females were slightly more numerous in each group (52% to 59%).^27^

**1.2.3 Description of Interventions**

Ardalan *et al. *used a community participation approach to preparedness and mitigation of flood disasters.^27^ Village disaster teams were developed, were trained, and then conducted training for local people that included identifying areas at risk of flooding, developing personalised plans, developing an early warning system and conducting evacuation exercises over three months.^27^ Eisenman *et al.* used a randomised trial to compare small group discussions with community health promotion officers lasting one hour (high-intensity intervention) compared to simply receiving written information (low-intensity intervention) for Latino residents of Los Angeles County, USA.^13^ A study of pregnant women in Japan offered six 15-minute disaster preparedness classes as part of normal antenatal classes in a cluster non-randomised trial.^24^ Participants in the intervention group were instructed about preparedness, communication and receiving medical attention in disasters. Controls received normal antenatal care.^24^ Two studies used games to educate participants about disaster preparedness. One used a specially developed board game, the Disaster Awareness Game, to inform school children about disaster preparedness for floods, hurricanes, volcanoes, landslides and mud flows.^31^ The intervention took place over two days and included discussion of the topics raised by the game. Tanes used an existing computer game, Beat the Quake, developed in California, which was designed to educate people about appropriate steps to take in the event of an earthquake.^18^^,^ 33

**1.2.4 Results**

Community flood risk preparedness programme participants were much more likely to have taken the recommended preparedness steps than people who lived in areas without the intervention.^27^ Adjusted odds ratios for the intervention group ranged from 6 to 50 for outcomes such as having a family preparedness meeting, risk mapping, having emergency supplies, having a plan for vulnerable people and having an evacuation drill. Control group odds ratios (comparing before and after) for the same measures ranged from 0.4 to 2.7.^27^ Latino people who took part in a community-based preparedness discussion with a health promoter and people who received written information made significant improvements in disaster preparedness according to a self-reported checklist.^13^ The *platica* (discussion) group was slightly better prepared for ten of 13 measures before the intervention. Overall preparedness score for the written information group increased from 29% to 52%, and for the *platica* group increased from 38% to 76%.^13^ In the Yasunari *et al.* study of disaster education with antenatal care, the intervention and control groups both showed non-statistically significant improvements in knowledge on all outcome measures, but no direct statistical comparison of the two groups was reported.^24^ The intervention group improved in knowledge of the emergency telephone line (69.7% to 82.8%), the disaster message board (51.5% to 67.7%), local alternative maternity clinics (52.5% to 68.7%), evacuation sites (47.5% to 67.7%) and being explain about own health status (86.9% to 97%).^24^ They also were more likely to have discussed communication plan with family (20.2% to 43.4%), be able to find out family contact methods (46.5% to 70.7%), take measures to prevent furniture turning over (33.3% to 48.5%) and objects falling (20.2% to 34.3%).^24^ The control group significantly improved in only taking measures to prevent objects falling (9.6% to 20.2%).^24^ The inclusion criteria for this study were altered *post hoc* when it was found that the demographics of the intervention and control groups (conducted on different hospital sites) were markedly different.^24^ Both game interventions led to increases in knowledge about hazards. The Disaster Awareness Game, played in schools on Caribbean islands, and supplemented with additional discussion, promoted increases in knowledge about all the natural hazards presented, as well as increases in knowledge about how to prepare, evacuate and recover from disasters. No statistical comparisons were made of the before and after comparisons, however. Among St. Vincent and the Grenadines participants, knowledge (measured by game score) of the hazard of flooding increased from 65% to 87%, and hurricanes from 65% to 83%.^31^ Among Turks and Caicos Islands participants, knowledge for the five tested hazards also increased (floods, 69% to 75%; hurricanes, 75% to 83%; volcano, 54% to 80%; landslide 51% to 92%; mudflow 43% to 83%).^31^ The Beat the Quake game increased knowledge about how to respond to an imminent earthquake according to scores on multiple choice questions, compared to not playing the game.^18^^,^ 33 The study evaluated more complex variations of the game involving comparing goal-setting by researchers or participants themselves, but no significant differences between these variations was noted.^18^^,^ 33

**1.3 Communication to Promote Preparedness for Man-made Disasters**

**1.3.1 Setting**

One study of communication for preparedness for man-made disasters was found.^12^

**1.3.2 Participants**

The participants who are relevant to this review were 421 people who attended voluntary clinics to receive potassium iodide (KI) prophylaxis and information, and 286 individuals who did not attend these clinics, all of whom lived within 10 km of a nuclear power plant.^12^ The participants had themselves chosen whether to acquire potassium iodide, and therefore the characteristics of the two groups were likely to be systematically different.

**1.3.3 Description of Interventions**

The New Jersey Department of Health and Senior Services organised distribution of KI to protect against thyroid cancer in the event of a nuclear or radiation incident. Voluntary clinics offered the pills and information to people within a 10km radius of a nuclear power plant. Information was written at Flesch Kincaid Grade level of 6.5 and pilot tested.^12^

**1.3.4 Results**

Individuals who received KI and information scored an average of 46% on a knowledge test, while those who did not receive KI or information scored an average of 15%.^12^ Due to the fact that the self-selection, it is difficult to evaluate the effect of the intervention, since those with better knowledge of radiation might have been more likely to seek KI, and therefore the intervention group might have had better knowledge anyway.

**1.4 Communications to Provide Early Warnings of Natural Disasters**

**1.4.1 Setting**

Three studies evaluated the effect of early warning of impending natural disasters. One evaluated the response to a warning of the Indian Ocean Tsunami on the island of Mauritius,^29^ and the second evaluated the effect of a warning of a cyclone on the east coast of India.^30^ A third study evaluated the response to an evacuation order during a wildfire event in San Diego, California, USA, in 2007.^16^

**1.4.2 Participants**

The survey in Mauritius reached 319 respondents (including 19 of whom outside the target area; proportion of each gender not reported) out of 1,484 attempted contacts.^29^ Sharma (2009) contacted 44 'decision-makers' (59% male) from individual households in Indian villages that were at risk of harm from a cyclone. Strawderman et al. interviewed 1,020 individuals (mean age 54 years; 37% male) who lived in a zone that was evacuated during the San Diego wildfire (of 12,204 telephone numbers attempted).^16^^,^ ^16^

**1.4.3 Description of interventions**

Perry evaluated the effects of the early warning of the Indian Ocean Tsunami on the inhabitants of Mauritius.^29^ Perry describes how the local authorities learned of the tsunami from international news outlets and began to issue warnings to inhabitants and tourists during the time period that the tsunami effects were already being observed.^29^ This included the meteorological service contacting police, coastguard, local radio and television channels and directly phoning hotels to warn them of the imminent danger. Perry reported that the information issued may have understated the necessary action required by individuals.^29^ Sharma et al. presented a study of the public's response to a warning of a cyclone on the east coast of India that occurred in December 2003.^30^ The intervention was a technical report warning of the impending cyclone and the likely effects. This was disseminated from national to local level through several layers of government, translated into local languages at district level and locally disseminated orally. The warning itself covered a large geographic area and did not advise on whether evacuation was necessary. The decision to advise people whether to evacuate was made by administrators at a district level in light of the information provided. Strawderman et al. sought to evaluate a recently-installed public address system called 'Reverse 911', which automatically dials all telephone numbers in a specified area and delivers a recorded emergency message.^16^ The warning messages were also spread through television, radio, interpersonal communication and police.^16^

**1.4.4 Results**

Perry reported that the Mauritius Meteorological Service learned of the tsunami just over two hours before its first effects reached the island.^29^ At the onset of the wave effects, 10% of people knew about the expected tsunami, and by the time the effects finished, approximately three hours later, only 42% knew about the warning. Upon hearing of it, most people continued their normal business (64%), while only 25% took action to protect themselves and 15% did the opposite of what was advised and went to watch the waves at the coast.^29^ In the study of a cyclone in India 345 of the villagers reported that they evacuated voluntarily, 20% were 'forced' to evacuate and 46% did not evacuate.^30^ In the San Diego wildfire, 53% of residents in the evacuation zone received the 'Reverse 911' evacuation message, and 80% who received it evacuated, including 100% of those for whom evacuation was judged necessary.^16^ However, only 66% of those who received it reported the message to be the reason why they evacuated. Some people who did not need to evacuate did so anyway because of the message.^16^ Information acquired through the Internet had the highest correct rejection rate, and the lowest false alarm rate, suggesting that residents were able to judge their own situation most accurately using it as a source.^16^ In the Mauritian tsunami study and the Indian cyclone study there was a low level of compliance with the advice to take protective action against the imminent natural hazards. Factors relating to personal circumstances, beliefs and attitudes, societal response, and the characteristics of the authorities may have influenced whether individuals chose to evacuate.^30^ In the San Diego wildfire, 69% of people in the evacuation zone did evacuate, which is much higher than in the other two studies.^16^ This may relate to the characteristics of the disaster (fire), the setting (rural USA), the communication methods, or the society. The 'Reverse 911' alert was relatively widely received and acted upon by a majority of recipients. Use of the Internet allowed people to make even more personalised decisions.^16^ The studies were all conceived retrospectively and relied on recall by participants. Some studies also assessed aetiological factors, and though the studies focused on the intervention of the evacuation warnings, they were not under the control of the researchers. It is difficult to assess the effectiveness of early warning information whenever it is not necessarily correct to attribute all subsequent evacuations to the warning, since some may occur independently of warnings.

**2 Effect of Risk Communication Interventions to Improve Disaster Response**

**2.1 Communication to Promote Response to Infectious Disease Disasters**

**2.1.1 Setting**

Seven studies of communication interventions during infectious disease disasters were identified. One was in the UK,^9^ one was in Egypt,^26^ and five were in Asian countries: one in each of China,^25^ Thailand,^21^ Taiwan,^22^ Singapore^23^ and Hong Kong.^20^ Three studies took place in the context of the SARS epidemic,^20^^,^ 23 ^,^ 25 one during the 2009-2010 influenza pandemic,^26^ one during an avian influenza outbreak,^21^ one involved an epidemic of viral conjunctivitis^22^ and one was set during a small outbreak of meningococcal disease in a school.^9^

**2.1.2 Participants**

Farahat et al. studied 420 school pupils (mean age 16 years; 46% male) during the influenza pandemic.^26^ Olsen *et al. *reported a study of 200 participants (median age 50 years; 72% female) during an avian influenza outbreak in Nakhon Phanom, Thailand.^21^ Three SARS studies took place in Asia. Pang *et al. *reported outcomes for 1,860 people in China who were hospitalised with SARS infection.^25^ A study by Chan *et al. *included 122 (63% female) people in Hong Kong aged 55 years or over.^20^ The study by Karan *et al. *included 300 individuals (51% male) in Singapore, 61% of whom were aged between 20 and 39 years.^23^ During an outbreak of viral acute haemorrhagic conjunctivitis in Taiwan, comparisons were made between two interventions by directing public health messages at the inhabitants of two affected cities, Taipei (population 2,632,242) and Keelung (population 390,084).^22^ Schoolchildren were the population that was mainly affected (Taipei City had 277,159 schoolchildren and Keelung City had 41,244).^22^ During an outbreak of meningococcal disease in a secondary-level school in England, 88 school pupils (63% male) aged 16-17 years were surveyed (of 198 who were invited) to investigate their response to the communications.^9^

**2.1.3 Description of Interventions**

One study used an intensive health education programme consisting of lectures, role-play and group discussion aided by photos, posters, pamphlets and a 'data show' about influenza H1N1 held every other day in schools for two weeks.^26^ During the avian influenza outbreak, a multi-media response was conducted, including a telephone help line with prevention information, a web site, newspaper, radio and television information broadcasts, the distribution of education videos to health officers and a booklet sent directly to members of the public.^21^ During the SARS epidemic in Beijing, the health ministry announced the outbreak in a press conference on 20 April 2003.^25^ Subsequently there were four further ministry press conferences, nine municipal government press conferences, a billboard campaign, bus advertisements, banners, a daily two-hour educational television programme, a telephone help line, 6,672 SARS community seminars and delivery of 8,280,000 copies of educational materials to citizens.^25^ In Hong Kong, Chan et al. conducted a telephone intervention with pre-test and post-test surveys.^20^ The intervention was delivered by nursing students who were trained to gather information from the participants and deliver a tailored intervention that addressed any of the participants' misconceptions or information needs about SARS. Karan et al. sought to evaluate the national public health communication programme in their survey.^23^ A multi-media campaign was conducted that involved press conferences, print and television adverts explaining infection control measures, a dedicated television channel, web site, help lines, and a 'SARS kit' containing a thermometer that was posted to every home. The Singapore response also included fines and imprisonment for individuals who did not comply with quarantine, and fines for some unhygienic behaviours.^23^ In the outbreak of infective haemorrhagic conjunctivitis, in both Taipei and Keelung cities delivered public service messages and health education in schools, ran a telephone help line and provided the media with information.^22^ However, Taipei instigated further steps: On a single day, the Taipei mayor addressed the press, a letter from the department of health was given to children to give to their parents and SMS text messages with information and infection control advice were sent to all 2.2 million Taipei citizens with mobile phones.^22^ The Health Protection Agency (now Public Health England) undertook a communication intervention to inform pupils and parents about an outbreak of meningococcal disease and to arrange mass antibiotic prophylaxis for all pupils and staff.^9^ The outbreak became apparent on a Friday afternoon, and the Health Protection Agency (HPA) issued letters to parents and released a press statement for radio on the following Monday. The following day, a briefing was held prior to the mass distribution of antibiotics.^9^

**2.1.4 Results**

Participants in the school-based intervention by Farahat *et al.* demonstrated improved knowledge and practice outcomes.^26^ Significant positive changes in knowledge about the cause, source, transmission, symptoms, complications and treatment of pandemic influenza were reported. Positive changes related to self-reported behaviours were also noted for respiratory and hand hygiene, infection control, and self-care. Overall, the proportion with satisfactory knowledge increased from 43% before the intervention to 68% afterwards, and the proportion with good practice rose from 57% to 65%.^26^ Evaluation of the Thailand multi-media response to the avian influenza involved interviewing participants after the events and asking them about their practices before and after the intervention, and as such it may be prone to recall bias. All four knowledge outcomes were significantly improved (believing that it is safe to touch dead poultry 40% to 14%; that it is safe for children to touch dead poultry 23% to 5%; preparing raw poultry with other food 50% to 37%; safe to eat undercooked chicken or eggs 21% to 6%).^21^ Behavioural practices did not improve to the same extent, with only those touching dead poultry with bare hands dropping from 39% to 11%).^21^ During the SARS epidemic, the median lag time between developing symptoms and hospitalisation in Beijing reduced during the time period of the outbreak from six days between 5 March and 9 April, to five days between 10 April and 20 April, and two days between 21 April and 15 June.^25^ Following the tailored telephone intervention, Chan *et al.* reported improvements in paired before and after analyses for knowledge about two of five points of knowledge about SARS transmission, but no change for the other three.^20^ For each question, between four and ten people (out of 122) answered correctly before the intervention, but incorrectly after it.^20^ For knowledge about transmission by droplets, for example, 62 people answered correctly before and after the intervention and 28 people answered incorrectly beforehand, but correctly afterwards, 19 answered incorrectly both times and four answered correctly before, but incorrectly afterwards.^20^ It was notable that for some outcomes (e.g. for whether animals could transmit SARS), the vast majority of respondents answered incorrectly before and afterwards.^20^ Unpaired analyses of behavioural outcomes also showed changes, but largely in the opposite direction to that expected. Overall, participants less frequently washed hands with soap or after coughing/sneezing (reduced from 1.3 to 1.7 on a four point rating scale with 1 = very often and 4 = not at all), less frequently covered their mouth when coughing/sneezing (reduced from 1.3 to 1.9), and were less likely to use a surgical mask (1.3 to 2.2).^20^ The authors speculated that this might be because the SARS epidemic was abating at that time, and there may have been less concern by the time of the second survey, one week after the first. It is, however, possible that the intervention had undesirable effects. The intervention's content is difficult to appraise because it was non-standardised, being tailored to each individual by the interviewers depending on their apparent knowledge.^20^ The evaluation of Singapore's response to SARS compared time points during and after the epidemic. The authors reported that 90% sometimes or always washed their hands regularly during the epidemic, but 80% did so afterwards.^23^ The recommended practice of monitoring one's own temperature daily, which can be assumed to have a zero prevalence prior to the public health campaign, was 85% (always or sometimes) during the epidemic and 31% afterwards.^23^ In the mass communication programme aimed at combating further spread of acute haemorrhagic conjunctivitis, school absenteeism and surveillance data were assessed.^22^ School absenteeism for affected pupils, desirable because it prevented further onward transmission of the virus, increased from 10% before the intervention to 62% afterwards.^22^ There was a fall in overall daily incidence from 0.093% to 0.056% following the intervention. The outbreak in Taipei lasted only 13 days, while that in Keelung lasted 34 days. The crude attack rate in Taipei was 2%, and that in Keelung was 14.9%.^22^ In qualitative responses to the Health Promotion Agency's response to an outbreak of meningococcal disease in a school, many pupils reported that the official communications had been too slow, because interpersonal communication had spread the news over the preceding days, but that this had been accompanied by misinformation and uncertainty.^9^ The report indicates that 74% of school pupils already knew about the meningococcal disease outbreak before information was released by the school and the Health Protection Agency.^9^ The Chan *et al.* uncontrolled before and after study during the SARS epidemic highlights a problem with uncontrolled studies: protective behaviours became less common during the period of the study, possibly because of a lessening concern about the disease as the outbreak abated.^20^ If the study had taken place earlier in the outbreak, it might have detected increases in protective behaviours as concern was rising, and might have attributed the improvement to the intervention.

**2.2 Communication to Promote Response to Natural Disasters**

**2.2.1 Setting**

One study relating to the response to natural disasters was identified,^17^ relating to the wildfire event in San Diego, California, USA, in 2007 that was also studied by Strawderman et al.,^16^ described above (Early Warnings).

**2.2.2 Participants**

Sugerman et al. conducted telephone interviews with 1,802 adults (of 18,687 calls made).^17^ Ten percent of calls were conducted in Spanish. The most frequent age range was 35-64 years, the study sample was older than the general population, and 50% of the sample were male.^17^

**2.2.3 Description of Interventions**

The health messages promoted by the San Diego County Health and Human Services Agency and American Heart and Lung Association during a three week multi-media campaign that used television, radio, newspapers and the Internet were evaluated.^17^ They advised residents to stay indoors, drive with all windows closed, run air conditioners on recirculate, keep home windows closed, use HEPA air filters, only exercise indoors, wet ash before clean-up, use N95 respirators during clean-up, limit activities to what is absolutely necessary, boil tap water before drinking and drink bottled water.^17^

**2.2.4 Results**

The messages reached 88% of respondents, with television being by far the most common medium (77%) followed by radio (7%).^17^ Following the warnings, participants spontaneously recalled being told to stay indoors (68%), to keep windows closed (18%) and only to exercise indoors (11%), but eight other messages were recalled by less than 5%.^17^ Fifty-nine percent stayed inside most of the day, 76% kept windows closed, 88% did not participate in outdoor sports, 76% wet ash during cleanup, 16% used home air conditioning, 10% used HEPA air filters and 8% wore a N95 mask.^17^ It is possible, however, that residents might have engaged in some of these precautions independently of the media campaign.

**2.3 Communication to Promote Response to Water Supply Disasters**

**2.3.1 Setting**

Four studies of the response to water supply disasters were found: One in Haiti,^32^ one in the UK^8^ and two in the USA.^15^^,^ 19 The Haiti study followed major flash floods due to Tropical Storm Jeanne.^32^ The study in the UK occurred in the context of major floods in Gloucestershire, England, that caused 140,000 homes to lose their water supply and subsequently be supplied with untreated water.^8^ The two USA studies took place in Boston, Massachusetts, after the collapse of a major water tunnel that carried water to 2 million individuals and residents were supplied with untreated water.^15^^,^ 19

**2.3.2 Participants**

The Haitian study interviewed 100 families who had received education and a water purification product following a natural disaster.^32^ Following the water crisis in Gloucestershire, 159 individuals (of 1,000 invited) who lost water supply were interviewed (40% male).^8^ The sample was slightly older on average than the local census (at least partly due to the absence of children from the survey). In Boston, Galarce et al. analysed surveys from 267 people who were affected by the water crisis (48% male; modal age 30-44 years).^15^ Participants were recruited from a research database (78%) and purposefully recruited from affected areas (22%).^15^ Wang et al. surveyed 525 adults (31% male; modal age 25-34) who were opportunistically recruited from hospital clinics and waiting rooms.^19^

**2.3.3 Description of Interventions**

In Haiti, community demonstrations were given of how to use PuR®, a proprietary water flocculation and purification treatment, three to six weeks after Tropical Storm Jeanne.^32^ The floods compromised and contaminated sources of drinking water. The US Centers for Disease Control and Populations Services International (PSI) had collaborated on the development of this product. PSI and the community leaders promoted, distributed and educated about the use of the water purification treatment.^32^ In Gloucestershire, a non-standard, locally produced 'Do Not Drink' notice was delivered to every affected household before untreated water was supplied to households, seven days after they lost all water supply.^8^ After a further seven days, a notice changing advice to 'Boil Water' was also delivered. Finally, a 'Water Safe' notice was issued. The local authorities communicated primarily through these notices, but local media also disseminated information.^8^ Galarce *et al.* described the issuing of a boil water notice by the Water Authority and dissemination by the Mayor's office, Boston Public Health Commission, schools, businesses, faith-based organisations, police, newscasters, the Centers for Disease Control and Prevention Health Alert Network, reverse 911 systems, phone calls, the Internet, by vehicles with 'bullhorns' and by other emergency management systems.^15^ The extent to which each was used was not described. Wang et al. investigated the same event and issuing of a boil water notice, but did not describe its dissemination.^19^

**2.3.4 Results**

Thirty-seven of the 100 Haitian families reported treating their drinking water before the floods. Following the flood and intervention, 58 used the product while other families used a variety of other reliable and unreliable methods.^32^ Knowledge about how to use the treatment was high: only two opened the packet using their teeth, 83 knew how much water to mix a sachet with, 88 knew how long to mix for, 79 knew how long to let the water stand for and 80 knew to put the flocculated waste in the latrine.^32^ Seventy eight answered all questions correctly. Use of the water treatment was not well sustained, however. Only 22 families had PuR®-treated water at the time of the interview, and only a few had used it often during the few weeks between distribution and survey. The authors suggested that this may have been because sample sachets were distributed without households knowing how to access further supplies.^32^ In the Gloucestershire water crisis, 89% received advice about the 'do not drink' notice, 71% about the 'boil water' notice and 88% about the 'water safe' notice.^8^ However, only 42% used the delivered notice for information during the 'do not drink' stage, 36% used it during the 'boil water' stage and 35% used it when the 'water safe' stage was reached. Local radio was more widely used at all stages. Questioning about respondents' knowledge revealed misunderstandings: Knowledge of the correct action during both the 'do not drink' and 'boil water' stages was low (23% and 27%). Compliance with the advice was not complete, and some people who did avoid drinking tap water continued to use it for brushing teeth and preparing food, which was against the advice. During the 'do not drink' stage, 9% of people drank un-boiled tap water, 16% brushed teeth with it and 21% prepared food with it. Many people used boiled water against advice during the 'do not drink' notice for food preparation (47%), to brush teeth (38%) and to drink (hot, 42%; cold 21%). When the advice was changed to 'boil water', 42% prepared food with, 38% brushed teeth with, and 29% drank un-boiled water.^8^ In the Boston water crisis, Galarce et al. found that 12.5% of people drank un-boiled tap water, 78% of respondents followed the advice to flush the cold water tap for 1 minute following the resolution of the water problem, and 59% of respondents flushed the warm tap for 15 minutes.^15^ The message successfully reached 89% of the sample on the first day of the crisis.^15^ The Wang et al. study reported that only 2.4% of their sample drank un-boiled tap water, and their data indicate that 74% of their sample knew about the crisis on the first day.^19^ Their survey reported 47% using bottled and boiled water, 41% using bottled water only and 9.6% using boiled water only.^19^ The study of water treatment in Haiti suggested that all households used some water treatment method following the floods, and that the participants were aware of how to use the water treatment appropriately.^32^ The study did not explicitly explore what happened when PuR® water treatment was not being used, so it is not clear whether alternative appropriate methods were being used or whether untreated water was also consumed. The intervention to disseminate information in Boston appears to have been on a larger scale than that used in the Gloucestershire crisis. The study following floods in England demonstrated misunderstandings by those affected and only moderate compliance with the recommended actions.^8^ The USA studies both showed higher compliance with advice, though it is worth noting that the Boston crisis lasted only 4 days, while that in England lasted up to 17 days for some residents.^8^^,^ 15

**3 Effect of Risk Communication Interventions to Improve Disaster Recovery**

**3.1.1 Setting**

One study took place following the 11 September 2001 New York terrorist attacks,^14^ and two studies took place following Hurricane Katrina in New Orleans.^10^^,^ 11

**3.1.2 Participants**

Media campaigns were conducted with the populations of New York and New Orleans as their intended audience. The monthly calls to the New York helpline, LifeNet, from November 2000 to December 2002 were enumerated.^14^ Similarly, calls to the New Orleans Crisis Line were counted (total 29,659 in the study period).^10^ In the second New Orleans study, 968 adult African Americans were interviewed during and after the media campaign.^11^

**3.1.3 Description of Interventions**

In New York, a mass media campaign was conducted using print, television, radio and other media (mainly fliers and billboards) between September 2001 and December 2002 with the aim of informing the city's population of the availability of a help line for mental health problems, which could provide advice and refer callers to medical services where necessary.^14^ In the context of this review, calling the help line was regarded as a behavioural outcome. Following Hurricane Katrina, a media campaign ran for 11 weeks in 2006 on four radio stations targeted at African Americans.^10^^,^ 11 Messages were broadcast five times each weekday on four radio channels. Five different messages with a focus on stress and depression were played. The messages promoted preventive behaviours (normal productive routine, social and physical activity and working to resolve conflicts), and information about an existing telephone help line that provides information and referrals for physician support, counselling and crisis intervention. A comparison group was made by comparing the number of calls to another phone number for the same help line that was not promoted in the campaign.

**3.1.4 Results**

In New York, major increases in advertising activity appeared to be accompanied by increases in call volume to the help line.^14^ Prior to the attacks, between 2,000 and 3,000 calls were made each month. In the month of peak advertising expenditure (September 2002), 9,000 calls were made (though this does coincide with the anniversary of the event).^14^ Similarly, in New Orleans, the number of calls to the advertised help line increased from a mean of 100 each day, to 125 during the campaign and 134 each day after the campaign.^10^ In the second study of this intervention, no change in combined post-traumatic stress disorder (PTSD) understanding, preventive behaviours (keeping routine, monitoring stress, talking with others, being productive and problem-solving) or screened incidence (indicated by avoidance, losing interest, isolation, losing hope, being jumpy and having difficulty sleeping) was found when comparing surveys taken before and during the campaign. A regression analysis incorporating the date of interview relative to the commencement of the programme showed an increase in PTSD beliefs/understanding and preventive behaviours, but no change in PTSD incidence.^11^ These interventions appear to have been effective at promoting the behaviour of phoning a help line for mental health problems following disasters. The campaign in New Orleans may have improved understanding and knowledge. The lack of change of PTSD incidence may be due to ineffectiveness of the intervention or due to a lack of sufficient time or power to detect any change in incidence.

**4 Risk of bias in included studies**

We used the Cochrane 'risk of bias' tool, which is designed for use with randomised control trials. Where a category was not applicable to the study design, 'Unclear risk' was chosen.

**4.1 Allocation (selection bias)**

Only one study was identified that allocated individuals randomly to one of two interventions.^13^ In that study, by Eisenman *et al.*, the allocation method and concealment were described.^13^ The Tanes *Beat the Quake *study used an unstated method of randomisation to allocate group time slots, not individuals.^18^^,^ 33 It was also not stated whether allocation occurred before or after the time slots had been selected by participants. The randomisation of groups increases the risk of selection bias. Virtually all the other controlled studies compared two different areas, or two groups of areas. Usually this was for practical reasons, and randomisation may not have been possible. For example, Ardalan *et al.* allocated intervention or control status to 31 villages and later adjusted analyses for possible confounders.^27^ The authors chose the allocations, increasing the risk that any differences in outcome between the intervention and control groups are due to something other than the intervention. Blando *et al. *studied two groups of people who had themselves chosen whether or not to receive the intervention several years earlier, which presents a high risk of selection bias.^12^ That study does not aim to be a straightforward evaluation of effectiveness, however, and the study should be interpreted in that light.

**4.2 Blinding (performance bias and detection bias)**

Blinding participants to the intervention that they are receiving is generally not possible in for the interventions identified in this review, although Eisenman *et al.* used interviewers who were blinded to intervention status in their randomised trial.^13^ No other studies mentioned blinding. Outcome assessment in before and after studies, controlled studies and interrupted time series would benefit from being conducted blind to the time point or intervention.

**4.3 Incomplete outcome data (attrition bias)**

The only randomised trial identified in this study had substantial attrition from both groups in the study, particularly from the high-intensity intervention.^13^ This could have affected the final results, since a per protocol analysis was presented. Ardalan *et al. *sought to avoid attrition by taking a further sample of participants for the post-intervention assessment.^27^ Many studies only conducted one interview with each participant, during which (if necessary) they asked participants to recall past as well as present knowledge or behaviours. Whilst avoiding attrition, this does increase risk of recall bias. In other studies, attrition was a significant problem. In the SARS intervention by Chan *et al.*, for example, one third of participants were lost to follow-up between two interviews.^20^ There was a risk of non-response bias in many of the studies, which was not often addressed. Rundblad et al. and Blando *et al.* both compared their study groups to the census data for the local population, but even this may not guarantee that people who are recruited are representative of the wider population in other characteristics.^8^^,^ 12

**4.4 Selective reporting (reporting bias)**

Some of the studies (mainly studies of preparedness) were clearly designed as prospective intervention studies. Numerous other reports sought to evaluate the response to disasters that had already occurred, in which case hypothesis could be formed cognisant of the general outcome of the interventions. Some of these may still have entailed a prospective collection of data. Others, however, could have generated hypotheses after data were already available. This is particularly possible for research into infectious diseases, given the presence of significant surveillance programmes in some countries. For instance, the use of 'lag time' in the SARS epidemic as an outcome, as reported in Pang et al., might carry some risk of being a *post hoc* analysis.^25^

## Discussion

**1 Summary of evidence**

Twenty seven studies of risk communication interventions for the mitigation of, preparedness for, response to, and recovery from disasters were included in this systematic review. The contexts, interventions and outcomes were diverse, and meta-analysis was not appropriate. Some disaster mitigation and preparedness interventions appeared to improve knowledge and behaviour relating to disaster risks. There was little robust evidence of the effectiveness of risk communication for disaster knowledge, behaviour and health outcomes in the response and recovery phases of disasters. One intervention was associated with an undesirable reduction in protective behaviours.

**1.1 Mitigation and Preparedness Interventions**

All four trials were found in this category. All mitigation and preparedness studies reported that the interventions that they reported were effective to some extent. Eastwood *et al.* reported a high level of intended compliance with public health advice before and after an intervention, but did not make statistical comparisons of the changes.^6^ Due to the already high compliance (over 90% for each measure), future studies in such contexts would therefore need to be very large to detect significant improvements. Dumais* et al.* reported significant improvements in knowledge for schoolchildren about influenza, which was intended only to facilitate further health education.^7^ The Roess et al. study of a monkeypox intervention found modest improvement in knowledge outcomes and some behaviours, but did not successfully affect some high risk behaviours.^28^ Assessing the longer term effect of the programme is likely to be difficult. Preparedness and mitigation for natural disasters was a relatively well-studied area with several prospective trials. Increased likelihood of behavioural preparedness was shown in controlled studies by Eisenman *et al.* and Ardalan et al..^13^^,^ 27 Games were used by two authors to increase knowledge, with apparent success.^18^^,^ 31 ^,^ 33 The effectiveness of the evacuation warnings is difficult to assess because of the many interacting factors in the situations. The Mauritius and India evacuations had relatively low voluntary compliance, whereas the Reverse 911 communication in the San Diego wildfires apparently provided a strong impetus for people to act.^16^^,^ 17 ^,^ 29 ^,^ 30

**1.2 Response Interventions**

Few of the response interventions were prospectively planned. The prospectively planned studies by Chan et al. and Farahat *et al., *though taking place in the context of ongoing disasters, were similar in character to the preparedness interventions for infectious diseases.^20^^,^ 26 Farahat et al. demonstrated improvements in knowledge and behaviour over three months, while Chan *et al.* reported a negative change for behaviour one week after their intervention.^20^^,^ 26 Attributing causality in the absence of a control group and in the context of widespread public and media interest in the topic of concern is difficult, and limits the value of these studies. All the other studies attempted to evaluate an ongoing or past risk communication response to a disaster. In these contexts, too, there are many factors that affect interpretation, including many other non-communication interventions that take place concurrently. The studies of the UK and USA water crises demonstrated that the communications to alert the public to the need to take action to avoid using contaminated water were successful in reaching almost all affected, but that in spite of this reach, a significant minority of people either did not understand the advice given or chose not to follow it.^8^^,^ 15 ^,^ 19 In Haiti, despite a high level of knowledge, the water treatment was apparently not used all the time.^32^ The factors that affect decisions about whether to comply with the advice may differ between these contexts.

**1.3 Recovery Interventions**

Three recovery interventions were found, including two studies of the post-hurricane Katrina response in New Orleans.^10^^,^ 11 Use of the mental health help lines in New York and New Orleans increased with promotion.^10^^,^ 11 ^,^ 14 Serial surveys of the audience in New Orleans suggested no change in one analysis, and changes in knowledge and behaviour, but not incidence of PTSD in a different analysis.^10^^,^ 11

**1.4 Disaster-specific Interventions**

We grouped the disasters primarily by phase and secondly by disaster type. It is possible, however, that there are important lessons for disaster types that transcend phases. We therefore also considered these reports together by disaster type. There were eleven studies related to natural disasters.^10^^,^ 11 ^,^ 13 ^,^ 16 ^,^ 17 ^,^ 18 ^,^ 24 ^,^ 27 ^,^ 29 ^,^ 30 ^,^ 31 ^,^ 33 All five preparedness studies reported that their interventions (games, interactive discussion groups or teaching) were effective means of increasing knowledge or preparedness behaviour.^13^^,^ 18 ^,^ 24 ^,^ 27 ^,^ 31 ^,^ 33 The only randomised trial identified for this review provided evidence in favour of a health promotion discussion group approach to general disaster preparedness over the simple provision of written information.^13^ In the three early warning studies of natural disasters diverse methods of communication were used. Reverse-911 appeared to be a promising method of communicating with at-risk populations, and people were able to validate their own risk using the internet.^16^^,^ 29 ^,^ 30 A study of a mass media campaign in response to a natural disaster suggested that some health messages were received and selectively acted on by the target population.^17^ Two studies of a recovery intervention to a hurricane suggested that a mass media campaign caused more people to contact a help line and that the media campaign might have increased understanding about post-traumatic stress disorder and might have been associated with increases in some preventive behaviours.^10^^,^ 11 It is interesting to note that natural disaster preparedness interventions, which authorities might wish to target at a whole population, mainly involved interpersonal communication and seemed quite resource-intensive. During disasters, responses were characterised by the need to communicate quickly, often by all available methods. The ability to directly communicate with people believed to be at risk, such as by the Reverse 911 alert, offers potential advantages over the use of third-party broadcast media, which usually targets a wider audience than those directly at risk.

There were ten studies related to infectious disease outbreaks, epidemics and pandemics.^6^^,^ 7 ^,^ 9 ^,^ 20 ^,^ 21 ^,^ 22 ^,^ 23 ^,^ 25 ^,^ 26 ^,^ 28 A group education approach was effective in increasing knowledge and improving planned behaviour related to a disease in a village and in two school settings.^7^^,^ 26 ^,^ 28 In another school setting during an outbreak of meningococcal disease, a qualitative study suggested that formal communication was slower than informal electronic and telephone communication, resulting in incorrect information being shared by school pupils.^9^ Studies of telephone education related to infectious disease disasters reported inconsistent results. During a SARS outbreak, one study reported an increase in potentially harmful behaviours, whilst a study of a pandemic influenza intervention suggested increased willingness to comply with protective behaviours.^6^^,^ 20 Multi-channel communication interventions were used in four other studies of infectious disease disasters.^21^^,^ 22 ^,^ 23 ^,^ 25 One of these used mass SMS text messages to give infection control advice in one of two settings.^22^ It is not possible to draw firm conclusions about the effectiveness because of the study designs, but the results of Yen *et al. *suggested that the use of mass SMS text messages may have contributed to improved compliance with infection control procedures and faster resolution of the outbreak.

**2 Limitations**

**2.1 Issues relating to study design**

Almost all the studies took place in the context of group interventions. However, no studies took full account for this in their analyses. This is a major problem for many of the studies where interventions were allocated at the group level. Analysing group-level studies at an individual level without accounting for the similarity of individuals within groups results in the study having more power than it should, and causes estimates of effect size to be unduly precise.^34^ Most of the studies were controlled or uncontrolled before and after studies and few of these used appropriate paired analyses. Separating the effect of the intervention from outside influences in uncontrolled studies is very difficult, particularly in disaster situations. The suddenness of many disasters that require evacuation means that planning and conducting prospective studies is difficult. Some disaster risk communication interventions are, however, amenable to study by more reliable methods, including individual or cluster randomised trials. In places where disasters occur relatively often, it may be possible to plan prospective comparisons of putatively similarly effective communication methods. Ethical issues must be addressed when planning such studies, but in principle, preparedness studies in which the control group subsequently are offered the intervention, or response studies that compare two putatively similar methods of risk communication (where it is not standard practice to use both together) are likely to be ethically sound.

**2.2 Issues relating to intervention design**

We had anticipated that this review would identify and evaluate studies that employed modern communications, including Internet-based social media. However, no studies that met the inclusion criteria tested these methods as an intervention. Conventional web sites were used in some organisational communications. Many complex interventions that occurred in response to disasters used many methods of communication at once and did not describe in detail how each was used. Two-way dialogue between the public and professionals was a feature of many preparedness interventions, but was not a common practice in responses to disasters, which tended to follow the traditional unidirectional model of disaster risk communication.

**2.3 Issues relating to the outcomes studied**

Due to the diversity of interventions and contexts, the outcomes for most studies differed. Knowledge outcomes were usually assessed in a survey following an information intervention that provided the basis for that knowledge. However, in the context of the early warnings, simply receiving the warning was assumed to be a knowledge outcome. Yet it is likely that simply receiving an early warning of an impending disaster is not the same as knowing and understanding the content of the message. Other outcomes could be valid and might be as important as knowledge and behaviour.

**3 Potential biases in the review process**

The use of the term *disaster* may have influenced the nature of studies included in this review, because events with similar cause but which do not meet the definition of disaster because they caused less severe disruption (possibly even because of a good public health response) will not have been included in this review. Valuable learning may therefore be lost because good practices that prevent an event overwhelming services and causing a disaster might not be found. Other studies may not have associated their research with a disaster, as they may be defined by the cause, and not the effect, of the disaster. In some reports, it was difficult to ascertain whether an event met the definition of disaster, since the local context may not be sufficiently described to indicate whether services were overwhelmed. This is particularly the case with preparedness and mitigation interventions as, for example, prevention efforts for many infectious diseases could be considered disaster mitigation, but may not have been presented as such in the published literature. The distinction between mitigation, preparedness and response to disasters sometimes seemed artificial, since it divided similar interventions that happened to take place before and during disasters (e.g. pandemic influenza)

## Conclusions

**Implications for practice**

Most of the studies included in this review reported improvements in disaster-related knowledge and behaviour. Due to the differences between the studies, it is not possible to conclude that one method of risk communication is superior to others. It is important to note the potential for harm from interventions, such as might have been the case in Chan et al..^20^

**Implications for research**

The major finding for future research from this study is that there is an absence of high-quality robust trials relating to disaster communication that should be remedied. Modern internet-based interactive social media present opportunities for risk communication, and may facilitate evaluation because it may be possible to invite recipients to complete knowledge or behaviour questionnaires, or even to request position-based information from mobile devices. Randomised trials (individual and cluster) of risk communication may have become more difficult in recent years because of the likelihood of 'contamination' between intervention and control groups due to sharing of information, which means that designing comparisons of information interventions will require careful planning to either prevent, or more likely, incorporate, the interpersonal information sharing that is a defining feature of modern communication methods.

## Competing Interests

One of the authors (Mike Clarke) is on the editorial board of PLOS Currents Disasters. The authors have declared that no other competing interests exist.

## Appendix 1

### PRISMA Checklist

 Download file

### Appendices 2-5

 Download file
